# Parametric emulation and inference in computationally expensive integrated urban water quality simulators

**DOI:** 10.1007/s11356-019-05620-1

**Published:** 2019-07-04

**Authors:** Antonio M. Moreno-Rodenas, Jeroen G. Langeveld, Francois H. L. R. Clemens

**Affiliations:** 1 Section Sanitary Engineering, Water Management Department, Faculty of Civil Engineering and Geosciences, Delft University of Technology, 2628 CN Delft, The Netherlands; 2 Department of Hydraulic Engineering, Deltares, 2600 MH Delft, The Netherlands; 3 Partners4UrbanWater, Javastraat 104A, 6524 MJ Nijmegen, The Netherlands

**Keywords:** Bayesian inference, Integrated catchment modelling, Dissolved oxygen simulation, Model emulation

## Abstract

Water quality environmental assessment often requires the joint simulation of several subsystems (e.g. wastewater treatment processes, urban drainage and receiving water bodies). The complexity of these integrated catchment models grows fast, leading to potentially over-parameterised and computationally expensive models. The receiving water body physical and biochemical parameters are often a dominant source of uncertainty when simulating dissolved oxygen depletion processes. Thus, the use of system observations to refine prior knowledge (from experts or literature) is usually required. Unfortunately, simulating real-world scale water quality processes results in a significant computational burden, for which the use of sampling intensive applications (e.g. parametric inference) is severely hampered. Data-driven emulation aims at creating an interpolation map between the parametric and output multidimensional spaces of a dynamic simulator, thus providing a fast approximation of the model response. In this study a large-scale integrated urban water quality model is used to simulate dissolved oxygen depletion processes in a sensitive river. A polynomial expansion emulator was proposed to approximate the link between four and eight river physical and biochemical river parameters and the dynamics of river flow and dissolved oxygen concentration during one year (at hourly frequency). The emulator scheme was used to perform a sensitivity analysis and a formal parametric inference using local system observations. The effect of different likelihood assumptions (e.g. heteroscedasticity, normality and autocorrelation) during the inference of dissolved oxygen processes is also discussed. This study shows how the use of data-driven emulators can facilitate the integration of formal uncertainty analysis schemes in the hydrological and water quality modelling community.

## Introduction

Integrated urban water quality modelling focuses on the joint simulation of processes driving pollution dynamics through the urban-river system (Muschalla et al. 2009; Rauch et al. 2002). These models jointly evaluate wastewater treatment processes, urban drainage and river dynamics, which usually generate a rapid escalation of complexity (Benedetti et al. 2013). The representation of all subsystems involved produces highly parameterised conceptualisations, requiring a large amount of data in the calibration process (Langeveld et al. 2013a). Additionally, the dynamics of interest often occur at very different time-space scales. For instance, urban combined sewer overflow (CSO) discharges have a characteristic timescale of minutes-hours whereas river dissolved oxygen dynamics exhibit hourly to monthly scales. Quantifying and analysing uncertainties in these platforms is hence required to avoid over-confidence in modelling results and to guide further model improvement (Deletic et al. 2012; Tscheikner-Gratl et al. 2017). However, the computational effort required is a severe limitation for the applicability of uncertainty analysis techniques for most real-scale integrated catchment modelling studies (Tscheikner-Gratl et al. 2019). Many uncertainty quantification strategies rely on intensive model sampling applications (Dotto et al. 2012). For instance, parametric inference schemes often require a large number of model evaluations (on the order of 10^4^–10^5^) to reach convergence. This hampers the use of formal uncertainty inversion methods in real-scale integrated urban water systems.

One approach to speed up convergence time is the use of optimised sampling schemes. For instance exploiting information from parallel model evaluations (Goodman and Weare 2010; Laloy and Vrugt 2012) or by using informed adaptive Markov chain Monte Carlo schemes (Hoffman and Gelman 2014). However, this still requires a prohibitive number of model samples, which often fall beyond the computational budget of most model users. A key strategy for the acceleration of model sampling is the use of data-driven or mechanistic model emulation, where a mathematical representation is used to approximate an interpolation map between a vector of parameters-inputs and the dynamic response of the simulator. Laloy et al. (2013) proposed a two-stage sampling scheme, first generating a rough estimate from a model surrogate and later from the simulator itself to perform parametric inference in a groundwater model. Carbajal et al. (2017) compared the performance of mechanistic vs. data-driven emulation for urban drainage simulators, concluding that in general a fully data-driven approach is to be preferred unless confronted with highly sparse training datasets. Yang et al. (2018) used a Gaussian process data-driven emulator to study parameter uncertainty in a semi-distributed hydrological model.

Data-driven emulators are constructed by drawing samples of the computationally expensive model at a selected number of input-parameter combinations. These samples are then used to build a database of input-output relationships. The emulator creates an interpolation between the two multi-dimensional spaces, thus allowing the fast estimation of the response when using a new parameter-input combination. Unfortunately, most mathematical structures used to emulate dynamic models (e.g. polynomial expansions, Gaussian processes) are sensitive to the dimensionality of the problem. The number of required samples to train the emulator increases non-linearly with the dimension of the input space (Xiu and Karniadakis 2002), thus reaching a point in which the construction of the emulator has an equivalent computational burden as using the simulator directly. Consequently, emulators often deal with a low number of static global parameters and a fixed time-window model output. The discretisation of input time series (as a parameter vector) can allow for the emulation of short time series (Mahmoodian et al. 2018) yet the length of the time series is limited to a few discrete steps, thus hampering its use in most cases. Hybrid strategies can be used to encode system knowledge in the data-driven emulator thus representing input dynamics. For instance, Moreno-Rodenas et al. (2018) presented a methodology to emulate hydrodynamic simulators (2D shallow water equations) under variations of parameters and time-dynamic rainfall inputs by encoding unitary response non-linearities in a polynomial expansion scheme. However, the generalisation of such input-parametric response emulation schemes to other variables (e.g. non-conservative water quality pollutants) still remains unaddressed.

Nevertheless, formal inference and intensive sampling techniques for uncertainty analysis are not being generally applied in integrated urban water quality modelling studies (Tscheikner-Gratl et al. 2019). This is primarily due to the high computational cost involved with such applications. The use of emulators can facilitate dealing with such large-scale modelling schemes, and thus further stimulate the consideration of modelling uncertainties in environmental studies. Moreno-Rodenas et al. (2019) presented an uncertainty analysis for a large-scale integrated catchment system for the assessment of water quality dynamics in the Dommel River (the Netherlands). The contribution of different uncertainty sources in dissolved oxygen depletion simulations in a highly urbanised river system was quantified. Forward uncertainty propagation showed that the use of prior knowledge (extracted from literature, measurements and expert elicitation) of the river physical and biochemical parameters captured roughly 70% of the statistical uncertainty in the simulation of dissolved oxygen dynamics. Performing inference directly on the original model structure is however prohibitive due to its high computational cost. An emulator structure was used to accelerate the model evaluation and thus updates system knowledge based on local observations. The development of this emulator and the inference of the model parameters are here presented.

This study discusses the application of a fully data-driven emulation scheme to accelerate the estimation of the dynamics of dissolved oxygen and river flow when varying a set of global river parameters. An emulator platform (polynomial orthogonal expansion) is created to represent an interpolation map between a set of river parameters (four water quantity and eight water quality process parameters) and the dynamic time series of river flow and dissolved oxygen concentrations at a location of interest. The training is performed by generating a database of model parameter to output relationships during the full year of 2012 (hourly frequency). The emulator is then used to implement a global sensitivity analysis and an inference scheme under various likelihood function conceptualisations. Consequently, this work shows that the use of a dynamic emulator scheme can facilitate the use of sampling intensive applications in large-scale simulators for water quality studies.

## Materials and methods

### The integrated catchment model

**Fig. 1:**
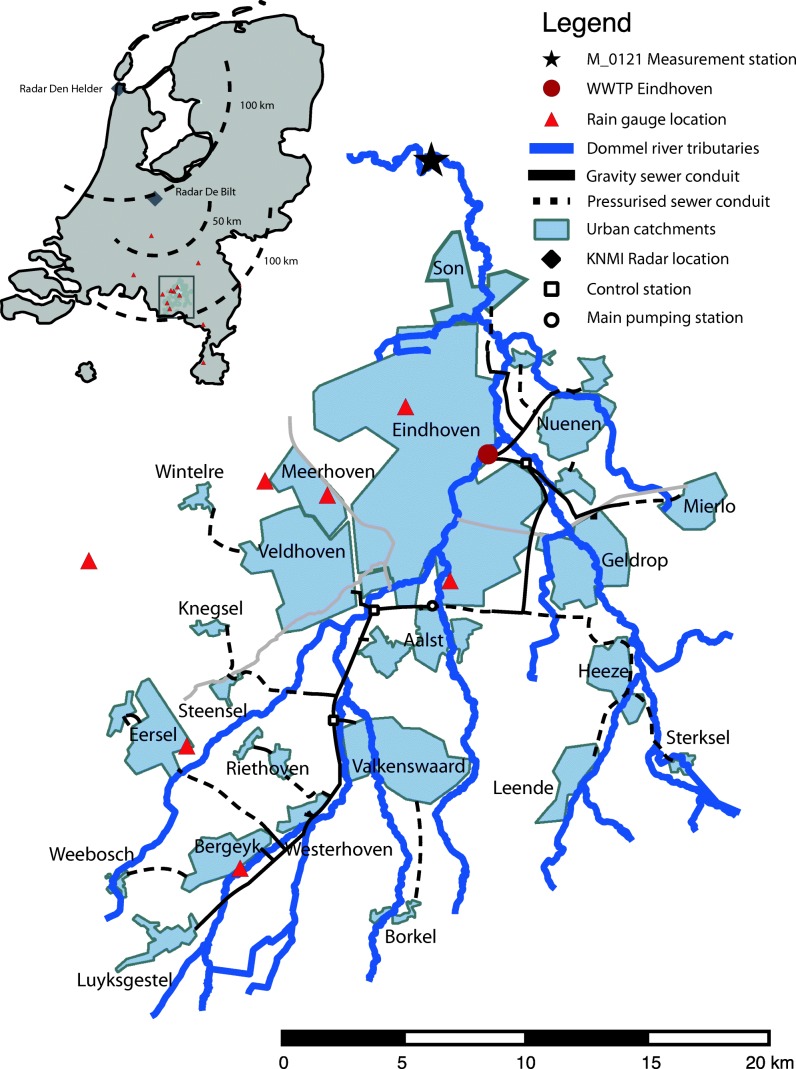
Urban water system of the Dommel

This modelling study targets the simulation of dissolved oxygen dynamics in the Dommel River. This is a sensitive stream located in the south of the Netherlands (Fig. 1). The river has a discharge between 2 and 20 m^3^/s, flowing through a mild-sloped lowland area. The river receives the discharge of 192 combined sewer overflow structures (CSOs) from several municipal urban drainage systems (connected urban area of ~ 4400 ha) and of a wastewater treatment plant (WWTP) of ~ 750,000 p.e. (population equivalent). High pollution loads from connected urban areas result in acute and chronic oxygen depletion events at the receiving water body. The integrated model accounts for the interaction between these three subsystems (river, urban drainage and WWTP). The river model is conceptualised as a tank-in-series hydrological scheme consisting 65 sections where the pollutant fluxes and transformation rates are computed (a conceptual scheme is presented in Annex A). The main river water quality processes are shown in Table 6 (Annex A). The WWTP was modelled through an a ASM2d (Gernaey et al. 2004) scheme representing three biological lines with primary clarifiers, activated sludge tanks and secondary clarifiers with a total capacity of 26,000 m^3^/h and a controlled bypass storm settling tank with a capacity of 9000 m^3^/h. Urban drainage flow was represented by 29 lumped rainfall-runoff and sewer transport schemes (Solvi 2006). Sewer water quality was represented by an influent generator at the WWTP (Langeveld et al. 2017) and by an event mean concentration multiplier at the CSO-receiving water links (Moreno-Rodenas et al. 2017b). The fully integrated model was implemented in the platform WEST (DHI). Further detail in the model and system characteristics can be found at Langeveld et al. (2013b) and Moreno-Rodenas et al. (2017a). Figure 14 (Annex A) depicts the model structure scheme. The river discharge and dissolved oxygen concentration were measured at a local station (M_0121, Fig. 1) with hourly frequency. Table 1 depicts some of the main characteristics of the observed data,

**Table 1:** Data characteristics at the observed period (2012)

Variable	Units	Location	Frequency	Mean	Std	Min	Max
River discharge	m^3^ · s^−1^	M_0121	Hourly	6.5	3.3	2.2	23
River DO	gO_2_ · m^−3^	M_0121	Hourly	6.9	2.1	0.3	11.6

### Dynamic emulator

A polynomial chaos expansion (PCE) structure (Xiu (2010)) was used to create an interpolation map between a set of model parameters and the model outputs of river discharge and dissolved oxygen concentration (at the location M_0121, Fig. 1) at hourly frequency during 1 year (01-01-2012–31-12-2012). PCE relies on fitting a series of orthogonal polynomials to the parametric-output multidimensional spaces. The model (*M*) consists of a large system of coupled differential equations, which represents the internal processes of the integrated water system to be emulated. An arbitrary time-dependent output state variable (*Y*_*sim*_***∈****ℝ*^*D*^) can be computed by solving the model:

1 $$ {Y}_{sim}=M\left({\boldsymbol{x}}_0,\boldsymbol{x},{\boldsymbol{\theta}}_M,{\boldsymbol{\theta}}_I\right) $$

given a set of *m* initial conditions (***x***_0_***∈****ℝ*^*m*^), a set of *r* dynamic inputs of length *F* (***x ∈****ℝ*^*rxF*^) and a group of global model parameters [***θ***_*M*_, ***θ***_*I*_], which in this case is decomposed as two parameter subsets; a group of global model parameters (***θ***_*M*_***∈****ℝ*^*S*^), which value is fixed by the modeller during the emulation and a subset of *P* global model parameters (***θ***_*I*_***∈****ℝ*^*P*^) for which the modeller seeks to emulate.

The emulator is composed of a series of *N* orthogonal polynomials (*ϕ*(***θ***_***I***_) ***∈****ℝ*^*Nx*1^) such that the value of the dynamic variable of interest (at a certain combination of emulated parameters ***θ***_*I*_) can be approximated:

2 $$ {Y}_{sim}\left(t,{\boldsymbol{\theta}}_I\right)\approx \phi {\left({\boldsymbol{\theta}}_I\right)}^{\mathrm{T}}\cdotp \mathbf{c} $$

where **c*∈****ℝ*^*NxD*^ is a matrix of coefficients which is calibrated based on samples drawn from the simulator, thus creating a mapping between the parameter and output spaces (*ℝ*^*P*^***→****ℝ*^*D*^). The training dataset is pre-computed by evaluating the model response at a number of *K* parameter combinations (***θ***_*I*_***=*** q_*i*_ for *i* = 1 … *K*). The training dataset is then used to calibrate the matrix of coefficients (**c**) such that:

3 $$ \left[\begin{array}{c}{Y}_{sim}\left(t,{\theta}_I={q}_1\right)\\ {}\vdots \\ {}{Y}_{sim}\left(t,{\theta}_I={q}_1\right)\end{array}\right]=\left[\begin{array}{ccc}{\phi}_1\left({q}_1\right)& \mathbf{\cdots}& {\phi}_N\left({q}_1\right)\\ {}\vdots & \mathbf{\ddots}& \vdots \\ {}{\phi}_1\left({q}_K\right)& \mathbf{\cdots}& {\phi}_N\left({q}_K\right)\end{array}\right]\cdotp \left[\begin{array}{c}{c}_1(t)\\ {}\vdots \\ {}{c}_N(t)\end{array}\right] $$

from which the polynomial values at each parameter sample, *ϕ*_*j*_(*q*_*i*_) and the model output *Y*_*sim*_(*t*, *θ*_*I*_ = *q*_*i*_) are known. A least squares approach was used to calibrate the set of coefficients **c** for each variable of interest. Then, Eq. (2) can be used to approximate the output variable at a new combination of emulated model parameters. Further information about the fit of polynomial expansions and the selection of orthogonal series can be found at Hadigol and Doostan (2018), Feinberg (2015) and Xiu and Karniadakis (2002).

Two polynomial expansions were used to emulate the modelled flow and DO dynamics at the receiving water body (1-year, hourly frequency series at the location M_0121, Fig. 1). The model response was emulated for variations of four (flow) and eight (DO water quality) model parameters respectively. The expansion was created using an orthogonal Legendre polynomial series (Gautschi 1994), truncated at 3rd order, and 200 training samples were drawn for both parameter spaces. Table 2 depicts the four river parameters for the emulation of flow and Table 3 shows the parameters for the emulation of DO along with their distribution (PCE training). Training samples were drawn using a Latin hypercube sampling (LHS) scheme.

**Table 2:** River hydrology parameter PCE training ranges (emulation) and prior distributions (inference)

Name	Units	Description	PCE training	Prior distribution
*n*	s · m^−1/3^	Manning roughness	~U(0.02, 0.15)*	~U(0.025, 0.12)
*k*_*z*_	–	Embankment slope multiplier	~U(0.3, 2)	~U(0.7, 1.3)
*k*_*W*_	–	River bed width multiplier	~U(0.3, 2)	~U(0.5, 1.5)
*k*_*surface*_	–	Rural flow input multiplier	~U(0.3, 2)	~U(0.7, 1.3)

*~U(*a*, *b*) refers to uniformly distributed probability density function between *a* and *b*

**Table 3:** River dissolved oxygen parameter PCE training ranges (emulation) and prior distributions (inference)

Name	Units	Description	PCE training	Prior distribution
*Kd*1	d^−1^	Decay rate for BOD fast	~U(0.3, 1)	~U(0.3, 0.8)
*Kd*2	d^−1^	Decay rate for BOD slow	~U(0.2, 1)	~U(0.2, 0.4)
*Vs*1	m · d^−1^	Sedimentation rate for BOD fast	~U(0.2, 40)	~U(0.5, 20)
*Vs*2	m · d^−1^	Sedimentation rate for BOD slow	~U(5, 100)	~U(10, 60)
*TKd*	–	Temperature coefficient for BOD oxidation	~U(1, 1.1)	~U(1, 1.1)
*TKL*	–	Temperature coefficient for reaeration	~U(1, 1.1)	~U(1, 1.03)
*TSOD*	–	Temperature coefficient for SOD	~U(1, 1.1)	~U(1, 1.1)
*VKL*	–	Velocity reaeration coefficient	~U(2, 8)	~U(2, 5)

The integrated urban water quality model depends on a large number of dynamic inputs and submodel parameters (e.g. urban drainage in-sewer parameters, WWTP parameters, rainfall inputs) some of which were of a stochastic nature. All model inputs and parameters were fixed to a deterministic realisation and only the emulated parameters were varied during the training database sampling.

Additionally, 100 and 50 random parameter samples were drawn independently for the flow and DO-simulated outputs respectively. This independent set was used to test the emulator’s performance by comparing the Nash-Sutcliffe efficiency (NSE) between the emulator vs. simulator output series at random parameter realisations.

The fitted coefficients of the polynomial expansion have a direct interpretability in terms of the sensitivity of the different parameters (Xiu 2010). Also, the emulator can be used to cheaply evaluate any combination of the parameters within the training parameter range, hence facilitating the application of sampling intensive system analysis tools. In order to describe the sensitivity of the studied model parameters, the Sobol global sensitivity analysis (Sobol 1993) was applied to the flow and DO river dynamics.

### Parametric inference

Prior knowledge of the river model parameters was encoded by means of independent uniform probability density functions. The parameter distribution ranges were defined based on literature values and expert criteria (non-formal elicitation). Tables 2 and 3 show the prior probability density function selected for each parameter.

This prior knowledge was updated by using an observation layout *Y*_*obs*_ ∈ *ℝ*^1*xL*^ for hourly measured flow and dissolved oxygen concentration at the outlet of the Dommel catchment during a period of approximately 7 months (15-Jan-2012–05-Aug-2012). The basic model observation layout was defined as:

4 $$ {Y}_{obs}=M\left({\boldsymbol{x}}_0,\boldsymbol{x},{\boldsymbol{\theta}}_M,{\boldsymbol{\theta}}_I\right)+Z $$

where *M* refers to the integrated catchment model. Initial conditions ***x***_0_ were computed using a warming up simulation period of 1 year for the WWTP initial conditions and a dedicated initialisation of the previous 15 days between 01-Jan-2012 until 15-Jan-2012 for the rest of the variables. The term *Z* refers to the residual structure between the simulated and measured series. This error term lumps measurement and model errors together. During the inference process, a probabilistic description of the model-measurement residuals *Z* is assumed a priori, and is later validated based on the posterior computed residuals. A common initial guess is to assume that residuals are independent, identically and Gaussianly distributed. This assumption leads to the following log-likelihood structure:

5 $$ \ell \left({Y}_{obs}|{\boldsymbol{\theta}}_I\right)\propto -\frac{1}{2}\log \left(\det \left(\boldsymbol{\Sigma} \right)\right)-\frac{1}{2}{\left({Y}_{obs}-M\left({\boldsymbol{\theta}}_I\right)\right)}^T\cdot {\boldsymbol{\Sigma}}^{-1}\cdot \left({Y}_{obs}-M\left({\boldsymbol{\theta}}_I\right)\right), $$

where **Σ** represents the residual covariance function, which in this case:

6 $$ {\boldsymbol{\Sigma}}_{\boldsymbol{Z}}={\sigma}_1^2\cdotp \mathbf{I} $$

being **I*∈****ℝ*^***LxL***^ the identity matrix and ${\boldsymbol{\sigma}}_{\mathbf{1}}^{\mathbf{2}}$ the constant variance of the residuals.

Flow dynamics are also known to render heteroscedastic error structures. This implies that residuals trend to be systematically larger when the discharge is larger. This is often encoded by assuming that the residual standard deviation follows a linear relationship with the simulated variable. This results in a log-likelihood function with the form described in Eq. (5) with the following covariance matrix:

7 $$ {\boldsymbol{\Sigma}}_{\boldsymbol{Z}\_\boldsymbol{het}}={\left({\sigma}_1+{\boldsymbol{Q}}_{\boldsymbol{F}}\cdotp {\sigma}_2\right)}^2\cdotp \mathbf{I} $$

where **I** ∈ *ℝ*^*LxL*^ is the identity matrix, ***Q***_***F***_ ∈ *ℝ*^1*xL*^ is the computed output time series; meanwhile, *σ*_1_ and *σ*_2_ are the hyperparameters of the error generating process.

Additionally, the inference of dynamic models often leads to autocorrelated residual structures. Previous studies in the hydrological literature have often taken this into account by the use of a discrete autoregressive model of order *p* (Bates and Campbell 2001), or as formulated by Schoups and Vrugt (2010):

8 $$ {\Phi}_p(B)\cdotp {z}_t\sim N\left(0,{\sigma}_1\right) $$

being ${\Phi}_B(B)=1-{\sum}_{i=1}^p{p}_i\cdotp {z}_{t-i}$ an autoregressive polynomial of order *p* for the residual *z*_*t*_, with Gaussian updates.

An equivalent formulation to account for a correlation structure was discussed by Honti et al. (2013) with the use of a bias description stochastic process *B* along with the error generating model (*Z*). If assuming a stationary continuous constant bias and heteroscedastic residuals, Eq. (5) defines the log-likelihood function, with a covariance matrix defined as:

9 $$ {\Sigma}_{{\left(Z\_ het+B\right)}_{ij}}={\left({\sigma}_1+{Q}_{\boldsymbol{F}\left({\boldsymbol{t}}_{\boldsymbol{i}}\right)}\cdotp {\sigma}_2\right)}^2\cdotp {\updelta}_{ij}+{\sigma}_3^2\cdotp {e}^{-\left|{d}_{i,j}\right|\cdotp {\tau}^{-1}} $$

which are the *i* and *j* elements of the covariance matrix ${\boldsymbol{\Sigma}}_{\left({\boldsymbol{Z}}_{\boldsymbol{het}}+\boldsymbol{B}\right)}\in {\mathbb{R}}^{LxL}$, with *δ*_*ij*_, the Kronecker’s delta, ${Q}_{\boldsymbol{F}\left({\boldsymbol{t}}_{\boldsymbol{i}}\right)}$ the expected flow (at time *t*_*i*_), *d*_*i*, *j*_ the distance in hours between *i* and *j* elements, *σ*_3_ a parameter of the stationary bias and *τ* an extra hyperparameter which drives a correlation exponential decay. Del Giudice et al. (2013) discuss that in practice the model effect and the bias descriptors can have a poor identifiability, thus inferring both, model parameters and bias hyperparameters, which often require assigning strong priors to the latter. Table 4 presents the prior distributions for the hyperparameters of the different likelihood distribution structures.

**Table 4:** Error model hyperparameters for the different hypotheses

Hyperparameter	Units	Description	Prior distribution
Flow i.i.d Gaussian			
*σ*_1_	m^3^/s	*σ*_1_, stationary standard deviation error	~U(0, 10)
Flow independent heteroscedastic Gaussian			
*σ*_1_	m^3^/s	*σ*_1_, stationary standard deviation error	~U(0, 10)
*σ*_2_	*m*^3^/*s*	*σ*_2_, stationary standard deviation error	~U(0, 10)
Flow AR(3) Gaussian updating			
*σ*_1_	m^3^/s	*σ*_1_, stationary standard deviation error	~U(0, 10)
*p*_1, 2, 3_	–	Autocorrelation coefficients p1, p2, p3	~U(0, 1)
Flow heteroscedastic normal error and exponentially correlated bias			
*σ*_1_	m^3^/s	*σ*_1_, linear intercept standard deviation error	~U(0, 10)
*σ*_2_	m^3^/s	*σ*_2_, linear slope standard deviation error	~U(0, 10)
*σ*_3_	m^3^/s	*σ*_3_, bias standard deviation	~U(0, 10)
*τ*	h	Tau, bias correlation exponential decay	~U(10, 80)
Dissolved oxygen i.i.d Gaussian			
*σ*_1_	mgO_2_/l	*σ*_1_, stationary error standard deviation	~U(0, 10)

Posterior samples were created using a Metropolis-Hasting algorithm (Hastings 1970; Metropolis et al. 1953). The joint prior probability distributions for the flow and dissolved oxygen river parameters were updated by drawing 50,000 samples from their posterior distribution by means of a Markov chain Monte Carlo sampling scheme (25,000 burn-in, 5 thinning). The Bayesian inference implementation was performed using the python probabilistic programming package PyMC version 2.3.6 (Patil et al. 2010).

Evaluating the likelihood distribution when using the full bias-description term involves inverting a covariance matrix of size *n*. In this case, this was prohibitively expensive when using the original measurement layout (*n* = 4892). Thus, a shorter period was used to test the inference of the bias description (26-Jul-2012–14-Sep-2012). In this case, only 4000 accepted samples were used (2000 burn-in, 2 thinning).

## Results and discussion

### Dynamic emulation of flow and dissolved oxygen concentrations

**Fig. 2:**
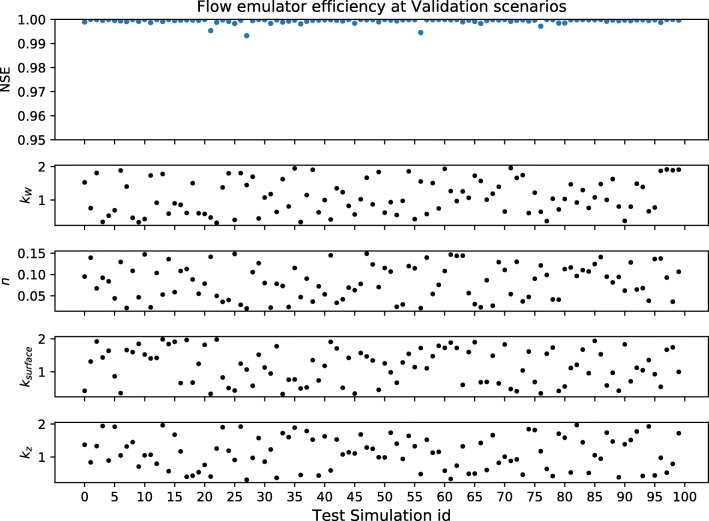
Nash-Sutcliffe efficiency (NSE) at the flow emulator vs simulation for a four-dimensional parameter space under validation conditions. The *x*-axis shows the 100 combinations of the parameter values (simulation id 0 to 99). Above, the NSE between 1-year hourly frequency time series simulated by the model and the emulator for each parameter combination

**Fig. 3:**
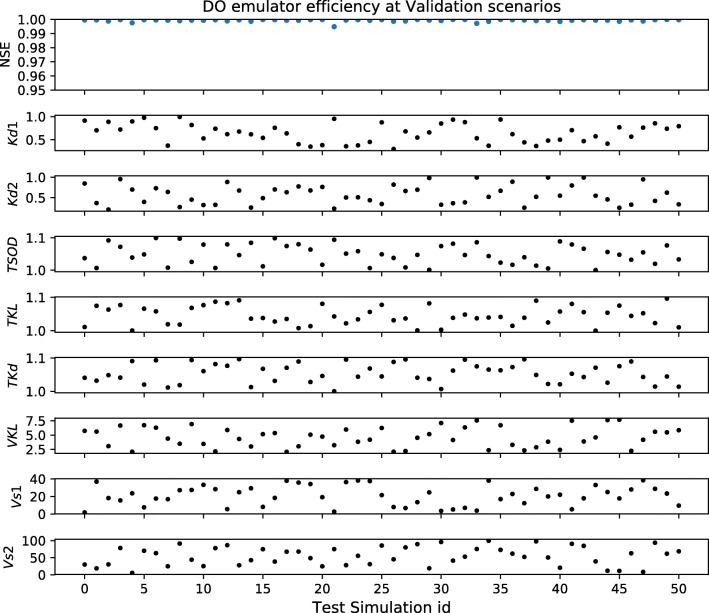
Nash-Sutcliffe efficiency of dissolved oxygen emulator vs simulator for an eight-dimensional parameter space under validation conditions. The *x*-axis shows the 50 combinations of the parameter values (simulation id 0 to 49). Above, the NSE between 1-year hourly frequency time series simulated by the model and the emulator for each parameter combination

The performance of the trained emulator to represent the integrated catchment model outputs (at new parameter combinations) was tested using an independent dataset. Figure 2 shows the Nash-Sutcliffe efficiency (NSE) between the emulated and simulated flow time series (1-year, hourly frequency) at 100 random parameter combinations for the flow emulator. Figure 3 shows the same test performed at 50 random samples drawn from the dissolved oxygen concentration emulation scheme. The performance of both emulator implementations is consistent across the parameter ranges and varies between 0.99–1 NSE. The observed performance during validation was considered sufficient for the substitution of the simulator by the emulator during the inference sampling.

Figures 17 and 18 (Annex C) show also a graphical comparison between the time series outputs from the emulator and the simulator at a series of random combinations of parameters independent from the samples drawn at the training dataset. Table 5 presents the computational effort required to sample from the original simulator, training and operation of the emulator. In this case, the computed average timings refer to a 2.2-GHz Intel Core i7 from mid 2014.

**Table 5:** Emulation vs. model computational effort for 1-year hourly frequency series (in seconds)

Sample	Flow	DO
Simulator sample	3300 s	3300 s
Training database (× 200 simulator samples)	660 × 10^3^ s	660 × 10^3^ s
Emulator training	14 s	61 s
Emulator sample	0.06 s	0.07 s

### Global sensitivity analysis of process parameters

**Fig. 4:**
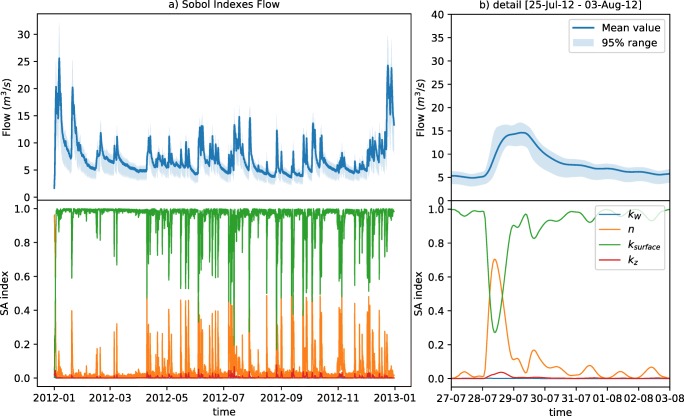
Sobol sensitivity indexes (first order) for the flow dynamics. Above, mean flow simulation and the 95% interval for the propagation of the parametric ranges. Below, sensitivity indexes for the four parameters. In the right, detail of the sensitivity during a medium-high intensity storm event (**b**)

**Fig. 5:**
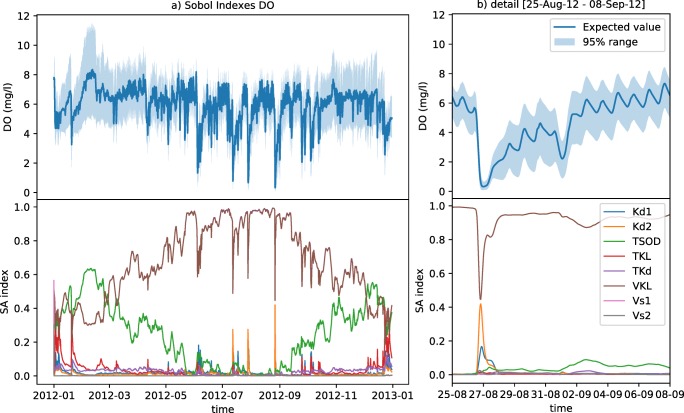
Sobol sensitivity indexes (first order) for the dissolved oxygen dynamics. Above, mean DO simulation and the 95% interval for the propagation of the parametric ranges. Below, sensitivity indexes for the eight parameters. In the right (b) detail of the sensitivity during a high intensity storm event

The emulator structure was used to estimate the sensitivity of the integrated catchment model outputs to variations of the river physical and biochemical parameters. Figures 4 and 5 depict the first-order Sobol sensitivity indexes from the prior distribution of parameters at the river flow and DO dynamics. Figure 4 shows the simulated flow level is highly sensitive on the parameter *k*_*surface*_ (which drives the river base-flow input) during dry-weather periods, whereas the manning’s roughness (*n*) becomes more sensitive during the rising limb of the hydrographs. *k*_*W*_ (multiplier for the river bed width) shows a reduced influence. *k*_*z*_ (a multiplier for the slope of the embankment) has a similar, yet less pronounced effect when compared with hydraulic roughness.

The results of the study of the sensitivity for the DO concentration simulation are shown in Fig 5. The parameter controlling the reaeration rate (*VKL*) dominates the dry-weather DO variability during summer times. This influence decreases during winter, where the temperature coefficient for the sediment oxygen demand (*TSOD*) becomes increasingly relevant. This has to do with the temperature inhibition model structure, which influences the oxidation rate of organic matter for temperatures differing from 20 °C (Annex A, Table 6). During oxygen recovery patterns, *TSOD* is also relatively relevant, since the sediment layer becomes the main oxygen sink (days after a large storm event). Sensitivity indexes do not show a consistent behaviour during acute oxygen depletion processes. Some depletion events, as the three occurring during July and September (also seen at Fig. 5b), present as dominant parameters *kd*1 and *kd*2 which are the oxidation rates for the two fractions of suspended BOD in the system. However, the events occurred in June and the three in October showed to be more sensitive to a different parameter combination as *TKL* or *TSOD*, which are related to temperature-driven reaeration or oxygen consumption. This is a good example of the complexity of the underlying process, in which interactions are highly dependent on the dynamic state of the system. For instance, if a storm event activates predominantly northern CSOs (Fig. 1), which are closer to the outlet of the catchment, there is less time for the degradation of suspended matter to occur than a more upstream storm process. Also, events in which the WWTP is the main source of discharge (and not CSOs), the settling facilities of the WWTP might lead to a lower sediment build-up in the river and thus increasing the relevance of suspended organic matter degradation.

### Parametric inference

**Fig. 6:**
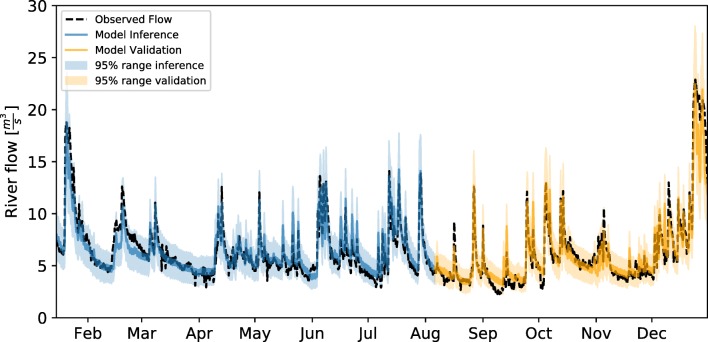
Posterior sample for the inferred flow dynamics between 15-Jan-2012 and 05-Aug-2012. In orange, the posterior distribution under validation conditions 05-Aug-2012 until 31-Dec-2012, in black observed flow at the station M0121

**Fig. 7:**
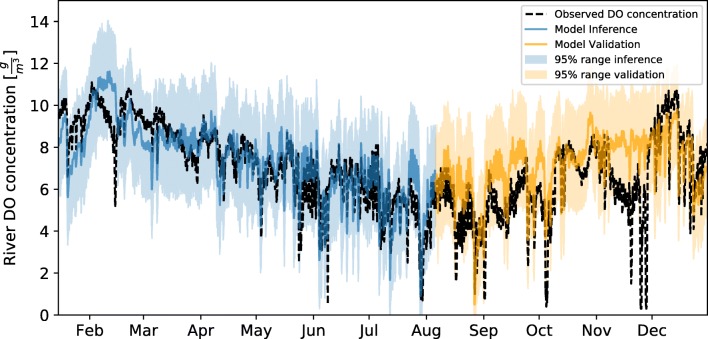
Posterior sample for the inferred dissolved oxygen dynamics between 15-Jan-2012 and 05-Aug-2012. In orange, the posterior distribution under validation conditions 05-Aug-2012 until 31-Dec-2012, in black observed flow at the station M0121

A local dataset was used to update the river parameters prior knowledge. The emulator allowed drawing fast samples (Table 5) from the posterior distribution of the parameters given several hypotheses for the error generating process (Gaussian, independent and homoscedastic for DO dynamics and Gaussian, independent and heteroscedastic for the river flow dynamics). Figure 6 displays the comparison of measured flow and the inferred mean model response. Also, a validation period (05-Aug-2012 until 31-Dec-2012) is shown. Intense dry weather periods induce a systematic overestimation of the flow as seen in July and in the beginning of September, this however is expected to have a limited influence in the water quality dynamics. The same comparison (measurement vs inferred and validation series) can be found in Fig. 7 for the simulation of dissolved oxygen. The general dynamics of DO are captured, especially the depletion processes, daily and seasonal variation.

**Fig. 8:**
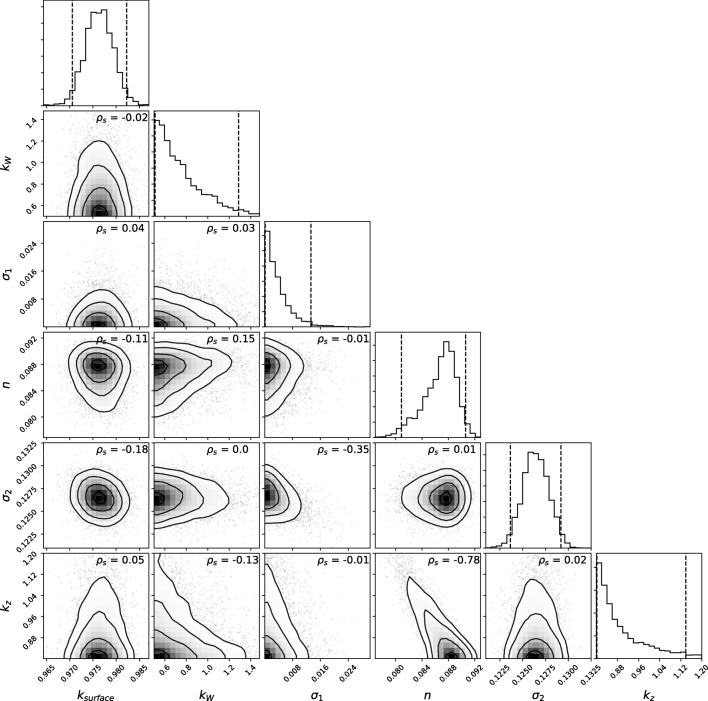
Posterior joint parametric distribution for the inference of the flow model parameters. ***σ***_**1**_ and ***σ***_**2**_ are hyperparameters of the selected error generation process (heteroscedastic, independent Gaussian). The spearman’s correlation coefficient (***ρ***_***s***_) is shown at each parameter couple

**Fig. 9:**
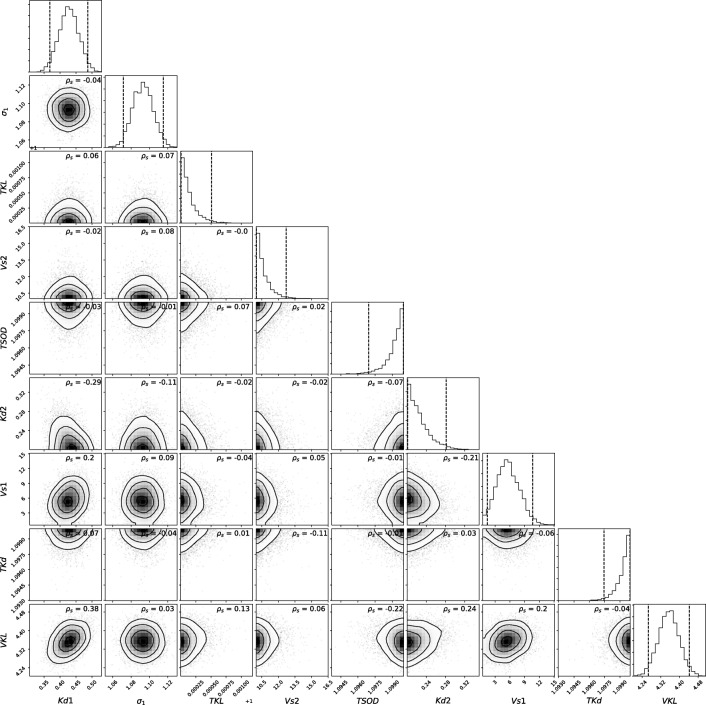
Posterior joint-parametric distribution for the inference of the water quality model parameters. ***σ***_**1**_ is the hyperparameter of the selected error generation process (independent, identically distributed Gaussian). The spearman’s correlation coefficient (***ρ***_***s***_) is shown at each parameter couple

The posterior probability density functions of the parameters for the water quantity and quality of the river section can be found in Figs. 8 and 9 respectively. The river variable *k*_*W*_ is poorly identified, which is denoted by the wide range of the posterior distribution (diagonal *k*_*W*_ at Fig. 8). This is also supported by the very low sensitivity of this parameter to the overall flow dynamics (Fig. 4). The rest of the parameters appear to be identifiable and are mostly mutually independent with the exception of a strong negative correlation between *k*_*z*_ and *n* (Spearman’s correlation coefficient, *ρ*_*s*_ =  − 0.78). Therefore, the joint inference/calibration of both elements is not recommended, since the provided observations lack sufficient information to identify these parameters independently. Further use of this model should therefore prioritize fitting *n*, since it exhibits a larger sensitivity than *k*_*z*_.

Water quality variables show a mostly independent joint posterior distribution with the exception of *kd*1 and *kd*2, which show a mild negative correlation (*ρ*_*s*_ =  − 0.29). This is explained by the fact that both parameters influence the same process (oxidation of organic matter) at two fractions of BOD for which DO measurements are probably insufficient to discriminate.

### Error-generating process and likelihood description

**Fig. 10:**
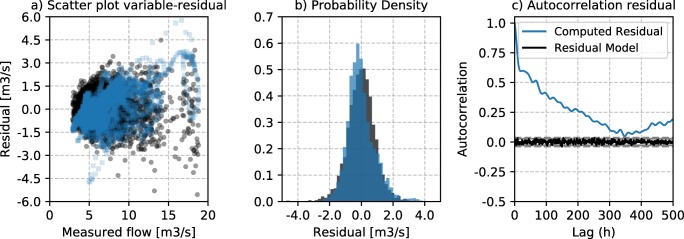
Residual structure at the flow posterior mean sample. **a** Scatter plot variable-residual showing the dependency of the variance. **b** The residual probability density. **c** The autocorrelation plot at different time-lags

Bayesian inference relies on the a priori definition of an error-generating process (Eq. 4), which constitutes the likelihood structure used during the inference scheme. The error-generating process is selected based on a series of hypotheses, which can be encoded by expert guesses on the behaviour of the system. Yet those assumptions are still a subjective exercise and its validity should be checked once sampled from the posterior distribution. In this case, the initial error generation process for both flow and DO series was conceptualized as an independent, identically distributed Gaussian distribution. The posterior distribution for the flow process revealed a dependency of the residuals and the inferred flow dynamics. Such phenomena is well described in the hydrological literature (Sorooshian and Dracup 1980) and was corrected by the use of a linear dependent standard deviation structure in the river flow error-generating process (Eq. 7). Figure 10 represents three relevant characteristics of the residual structure at the posterior samples of river flow, comparing the assumed error-generating process (in black) and the observed one (in blue). Figure 10a shows the heteroscedastic structure of flow residuals, Fig. 10b shows the comparison of the residual histograms and Fig. 10c shows the time-autocorrelation structure. It is apparent that the residual independency assumption is violated, since computed residuals present a strong time autocorrelation structure.

**Fig. 11:**
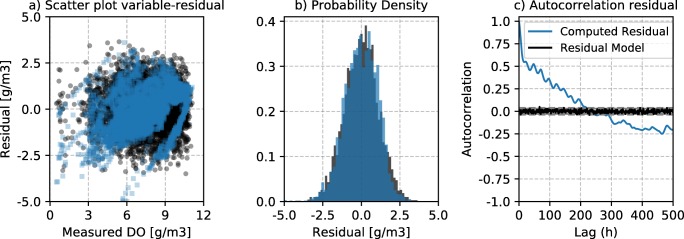
Residual structure at the dissolved oxygen posterior mean sample. **a** Scatter plot variable residual showing the dependency of the variance. **b** The residual probability density. **c** The autocorrelation plot at different time-lags

Fig. 11 shows the comparison of the computed residuals and the assumed error-generating process (independent, homoscedastic and Gaussian) for the dissolved oxygen in the river. The variance of the residuals is largely independent from the DO value. Also, residuals present a clear autocorrelation structure, albeit shorter than that of the flow inference.

The time autocorrelation structure in hydrological inference has been discussed in several studies. For instance, Kuczera (1983) applied an ARMA (autoregressive moving-average) model to represent an autocorrelated likelihood structure in a hydrological model. Bates and Campbell (2001) argued that ARMA structures lead to local minima, and AR (autoregressive) models of order p are to be preferred. Schoups and Vrugt (2010) presented the use of an alternative likelihood structure, which addresses several common issues; like the non-normality of residuals, variance non-stationarity and the temporal correlation of the residuals (captured by an AR(p) model). Yet all these three studies simulated catchment hydrological flows at daily scales. In this work, the measurement layout has an hourly time-step, since relevant processes occur at those scales. It is expected that the autocorrelation structure becomes stronger when dealing with shorter timescales. As seen in Figs. 10 and 11 the correlation is still around 0.5 at 50–100 h lag time. Honti et al. (2013) and Del Giudice et al. (2013) presented the direct encoding of a bias description process within the error model. This was applied to urban drainage hydrodynamic simulation with time-steps of 1–2 min, which present strong autocorrelation structures. The bias description can be conceptualised as function of different variables or inputs, yet in its basic form, it constitutes a Gaussian multivariate distribution with an exponential covariance structure as in Eq. 9.

The use of an AR(3) model, in this case, rendered an almost negligible effect of the autoregressive parameters of higher order than one (< 0.01), thus generating an equivalent AR(1) model. Measured series vs. inferred comparison and the residual structure can be seen in Fig. 15 (Annex B). Although the autocorrelation of residuals is better represented, the fit of the mean sample did not improve, rather was degraded through accounting for the autocorrelation term. This was also discussed by Evin et al. (2013), who showed that using AR(1) models for hydrological inference on the raw residuals can lead to strong interactions with the inferred parameters and degraded outcomes.

**Fig. 12:**
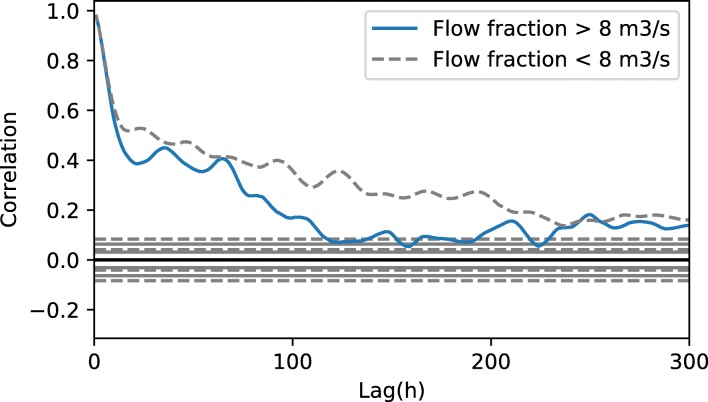
Autocorrelation structure for flow residuals by magnitude

**Fig. 14:**
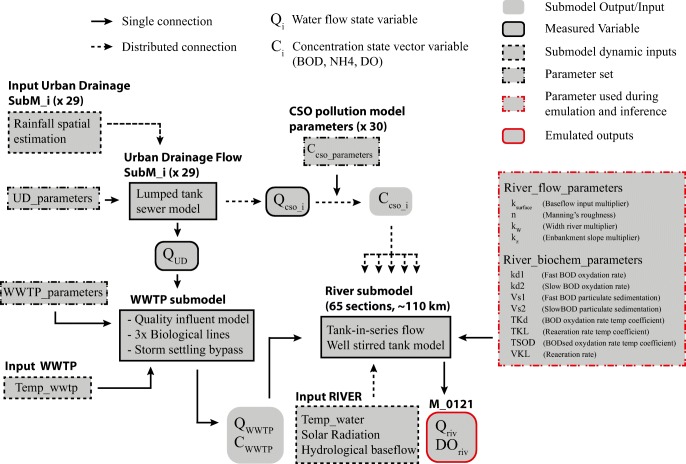
Model scheme

**Fig. 15:**
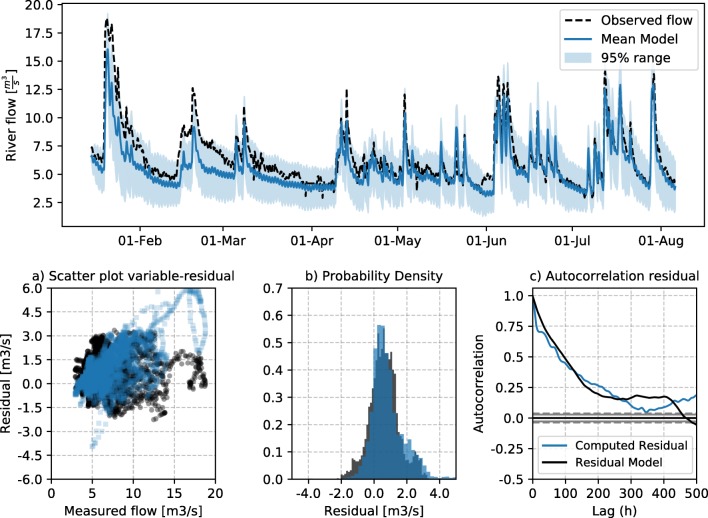
Autoregressive model order 3, comparison of measured and inferred dynamics and residual structure

**Fig. 16:**
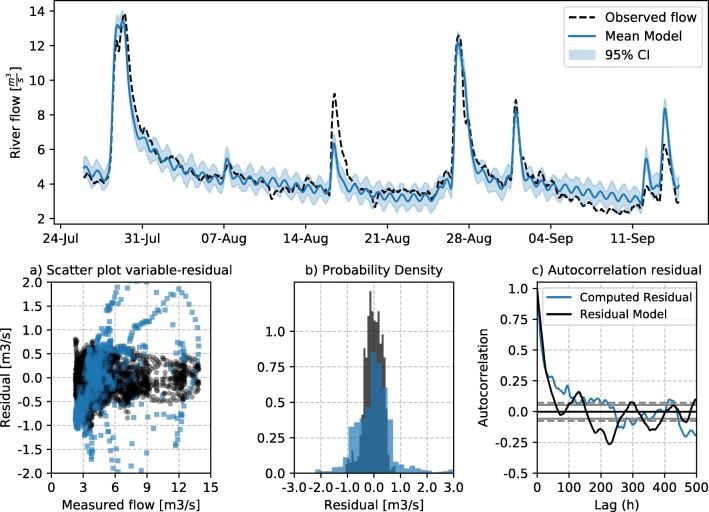
Bias description, comparison of measured and inferred dynamics and residual structure

**Fig. 17:**
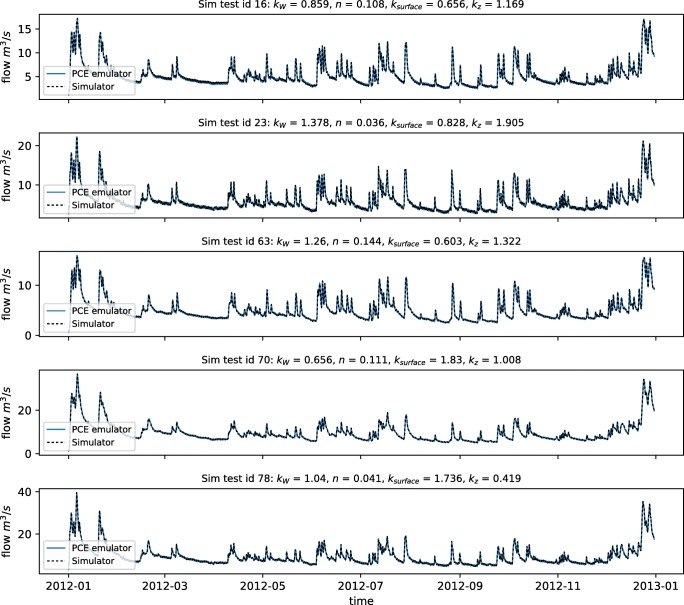
Emulator vs. simulator flow time series graphical comparison for different test parameter combinations

**Fig. 18:**
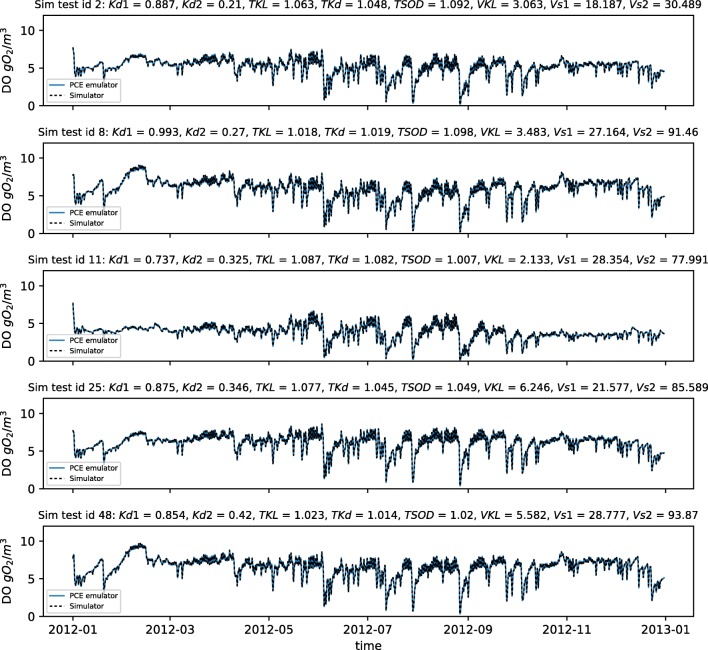
Emulator vs. simulator dissolved oxygen time series graphical comparison for different test parameter combinations

On the other hand, the use of a bias description as in Del Giudice et al. (2013) becomes prohibitive for long time series. This implementation requires the inversion of a covariance matrix *Σ* ∈ *ℝ*^*LxL*^ being *L* the size of the measurement layout. In this case, considering an hourly sampling layout during 15-Jan-2012 until 05-Aug-2012 leads to *L* = 4892 elements. Expected values of the decay parameter *τ* are likely to produce a highly sparse covariance matrix, thus sparse inversion optimisation could be applied (Betancourt and Alvarado 1986) yet intensive sampling for populating the posterior is still cumbersome. This large covariance matrix inversion renders the evaluation of the likelihood function computationally expensive, thus eliminating the benefits of the use of the dynamic emulator. A possible solution is to create a database of likelihood samples from the dynamic emulator which is used to build a second emulator linking the parameter space and the response of the likelihood function (Dietzel and Reichert 2014). An illustrative example of the residuals when using a bias description term is shown in Fig. 16 (Annex B) in which the inference was performed in a shorter time series (26-Jul-2012–14-Sep-2012). The description of the autocorrelation structure in the residuals did not allow for a better description of the process, or a better understanding of the parametric uncertainty. Ammann et al. (2018) recently studied the representation of autocorrelated likelihood structures with the conventional error models for hydrological applications. They discussed that the use of stationary autocorrelation models deteriorates the performance of the inferred model significantly (degrading even further when increasing the measurement layout frequency). They propose that the use of non-stationary autocorrelation schemes may overcome this problem, since hydrological models are expected to lose memory under storm events (thus dry-weather and wet-weather present different residual correlation patterns). This non-stationarity could not be found in this case, being the correlation structure in dry weather and wet weather for short scales (0–80 h lag) equivalent and the large-lag structures only slightly different (Fig. 12). Also, this is not expected to be applicable for DO series, where residuals are even less structured as in hydrological flow.

A strong autocorrelation structure is expected due to the nature of the process and the measurement layout. Both flow and dissolved oxygen concentrations present several dynamic modes, induced by storm events, daily fluctuation in the WWTP effluent and variation between dry-wet periods and temperature seasons. Small temporal shifts are expected due to model structural misfit (e.g. incorrect CSO timing in the urban drainage scheme or misrepresentation errors in rainfall data). The temporal shift will likely render strongly correlated residuals in time. Yet these time-shifts are of limited influence for the model application. The objective of the model is to represent dynamics of oxygen in a receiving water body for environmental policy assessment studies. These studies use metrics which lumps the time-dynamics, as frequency-duration-concentration tables (FWR 2012); thus, the exact timing of the oxygen depletion is not highly relevant, but rather the correct representation of the magnitude and duration of each event. Consequently, the stiff likelihood conditions required to construct formal inference schemes (as shown in this study) might not render the most adequate approach when dealing with the with long-term dissolved oxygen dynamic series (in which the system exhibit multitude of complex dynamic states). Approximated Bayesian computation (Toni et al. 2009) could be of interest by allowing defining metrics which attend to the relevant features of the dissolved oxygen output space (e.g. duration and magnitude of events, slopes of the depletion/recovery patterns, etc.). The selection of signature-based metrics are being increasingly used for the diagnosis of hydrological modelling studies (Kavetski et al. 2018; Vrugt and Sadegh 2013) and could also facilitate the identification and calibration of urban water quality dynamics.

## Conclusions

This study presents the emulation and inference of river flow and dissolved oxygen dynamics in an integrated urban water quality system. The simulator jointly evaluates wastewater treatment processes, urban drainage and receiving water quality processes. The use of an emulation scheme allowed accelerating significantly the approximation of the response of the simulator to variation of a set of global parameters. This facilitated the implementation of sampling intensive applications (e.g. sensitivity analysis and formal Bayesian inference schemes).

A polynomial orthogonal expansion emulator was fitted to represent flow and dissolved oxygen depletion for a 1-year-long time series (hourly frequency) under four and eight global parameters respectively. Two hundred model realizations sufficed to generate an acceptable interpolation in both cases. The emulator was validated using independent data, rendering a high-quality mapping between the parametric space and the dynamic response. This technique still exhibits severe limitations, like the impossibility to include large parametric spaces, dynamic inputs or non-smooth parametric to output relationships (e.g. bifurcation solution points).

The use of the emulator facilitated the computation of the sensibility of flow and DO dynamics to river parameters. Oxygen depletion processes exhibit a non-stationary dependency across storm events. In general, the reaeration rate showed to be the most relevant parameter during dry weather flow. Depletion of fast-slow biodegradable matter is often the responsible for the magnitude of the oxygen depletion event (attending only to the river process parameters). Meanwhile, oxygen recovery after a large depletion event is highly influenced by the sediment oxygen demand and reaeration processes, with a strong dependence in seasonality (temperature driven).

A set of observations of river flow and DO was used to update prior knowledge about the receiving water model parameters. This was achieved by performing an inference scheme on the emulator as a substitute of the simulator. Several hypotheses were used to define the likelihood structure. A homoscedastic, independent Gaussian distributed error was applied to the dissolved oxygen error process. Meanwhile, the flow residuals showed a heteroscedastic structure. Both inferred residual series rendered a highly temporally correlated structure, which violates the assumption of independence. The residual autocorrelation is related to the measurement layout frequency (hourly) and the nature of the simulated processes. Both flow and DO residuals are influenced by a strong memory effect, model structure-induced time shifts and input errors. Various formulations to deal with the residual autocorrelation or structural bias were tested. However, the inferred dynamics either deteriorated or did not improve. Detailed investigation on the effects of neglecting the correlation structure in the dissolved oxygen residual structure is still missing. Also, the use of alternative metrics for the inference of dissolved oxygen dynamics should be further studied.

The use of a dynamic emulation scheme allowed gaining insights on the underlying mechanistic relationships of the integrated urban water quality system. This can be easily extended to similar environmental modelling studies thus facilitating the application of sensitivity analysis, inference or calibration under long time series and low-dimensionality parametric spaces.

## Figures and Tables

**Fig. 13:**
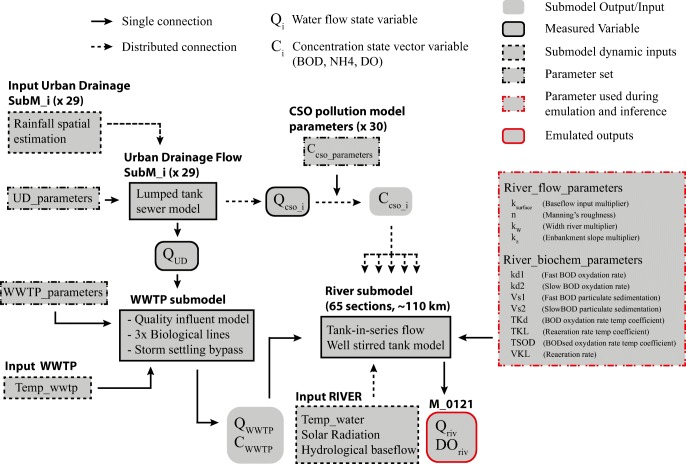
Tank in series river flow scheme

## Annex A: River model water quality processes

Table 6 presents the process matrix for the dissolved oxygen process routine at the river section. The model accounts for the transport and conversion rates of seven concentration state variables; BOD1 (biological oxygen demand of fast biodegradable suspended fraction), BOD2 (biological oxygen demand of slow biodegradable suspended fraction), BOD1p (biological oxygen demand of fast biodegradable particulate fraction), BOD2p (biological oxygen demand of slow biodegradable particulate fraction), BODs (biological oxygen demand at the sediment section), NH4 (ammonium) and DO (dissolved oxygen concentration).

**Table 6:** Process matrix for the river water quality model structure

State variable	BOD1	BOD1p	BOD2	BOD2p	BODs	NH4	DO	Rate
	gO_2_/m^3^	gO_2_/m^3^	gO_2_/m^3^	gO_2_/m^3^	gO_2_/m^3^	gN/m^3^	gO_2_/m^3^	
Process								
1a. Oxidation of fast-suspended fraction (BOD1)	−1						−1	${TKd}^{T_{wat}-20}\cdotp Kd1\cdotp BOD1\cdotp \frac{DO}{KO2+ DO}$
1b. Oxidation of fast-particulate fraction (BOD1p)		−1					−1	${TKd}^{T_{wat}-20}\cdotp Kd1\cdotp BOD1p\cdotp \frac{DO}{KO2+ DO}$
2a. Oxidation of slow-suspended fraction (BOD2)			−1				−1	${TKd}^{T_{wat}-20}\cdotp Kd2\cdotp BOD2\cdotp \frac{DO}{KO2+ DO}$
2b. Oxidation of slow-particulate fraction (BOD2p)				−1			−1	${TKd}^{T_{wat}-20}\cdotp Kd2\cdotp BOD2p\cdotp \frac{DO}{KO2+ DO}$
3a. Sedimentation of BOD1p		−1			+1			*Vs*1 · *BOD*1*p*
3b. Sedimentation of BOD2p				−1	+1			*Vs*2 · *BOD*2*p*
4. Oxidation of organic matter in the sediment					−1		−1	$TSO{D}^{T_{wat}-20}\cdotp {K}_{BOD}\cdotp \frac{ BOD s}{d}\cdotp \frac{DO}{KSO+ DO}$
5. Constant sediment oxygen demand							−1	$TSO{D}^{T_{wat}-20}\cdotp \frac{SOD}{d}\cdotp \frac{DO}{KSO+ DO}$
6. Nitrification						-1	−4.57	${TKnit}^{T_{wat}-20}\cdotp Knit\cdotp NH4\cdotp \frac{DO}{KNO2+ DO}$
7. Photosynthesis macrophyte							+1	${TKp}^{T_{wat}-20}\cdotp {k}_{prodM}\cdotp Io\cdotp \frac{MB}{d}$
8. Macrophyte oxygen consumption							−1	${TKp}^{T_{wat}-20}\cdotp {k}_{pcons}\cdotp \frac{MB}{d}$
9. Reaeration							+1	${TKL}^{T_{wat}-20}\cdotp VKL\cdotp \left( CS- DO\right)$

The river flow was conceptualized as a tank in series lumped scheme, in which each tank is a well-stirred reactor in which the biochemical processes take place (Fig. 13). The mass balance equation is computed at each node:

1 $$ \frac{d{V}_i}{dt}={Q}_{i{n}_i}-{Q}_{ou{t}_i}, $$

where *V*_*i*_ is the volume at tank *i* and ${Q}_{i{n}_i}$ the sum of inflows from CSOs, upstream sections or rural hydrology. ${Q}_{out_i}$ is the outflow from the tank at each instance and is computed as a free-surface flow following the Gauckler-Manning equation:

2 $$ {Q}_{ou{t}_i}=A\cdotp {R}_H^{\frac{2}{3}}\cdotp sl{p}^{\frac{1}{2}}\cdotp {n}^{-1}, $$

being *A* and *R*_*H*_ the area and hydraulic radius for a trapezoidal section with a given slope (*slp*) and bed Manning’s roughness (*n*).

Figure 14 depicts the whole integrated system scheme which comprises the urban drainage networks connected to the area of interest in the Dommel, the WWTP of Eindhoven and the river stretch considered in the water quality model along with the parameter and input model spaces.
